# Case Report: Primary graft failure due to a reversed lenticule in Descemet Stripping Automated Endothelial Keratoplasty

**DOI:** 10.12688/f1000research.123313.1

**Published:** 2022-09-27

**Authors:** Anahita Kate, Sayan Basu

**Affiliations:** 1 The Cornea Institute, L V Prasad Eye Institute, Vijayawada, Andhra Pradesh, India; 2 The Cornea Institute, L V Prasad Eye Institute, Hyderabad, Telengana, India

**Keywords:** Endothelial keratoplasty, Descemet stripping automated endothelial keratoplasty, inverted lenticule, graft adhesion, primary graft failure

## Abstract

**Introduction and importance**: This report details the clinical features and management in a case of Descemet stripping automated endothelial keratoplasty (DSAEK) which had primary graft failure (PGF) due to an inverted yet attached lenticule.

**Presentation of case**: A 66-year-old gentleman had poor visual recovery in the right eye after undergoing cataract surgery 12 years prior to presentation. The visual acuity was counting fingers and examination revealed endothelial decompensation. The patient underwent a DSAEK and postoperatively had a well attached lenticule. However, the cornea was edematous three weeks after the surgery and optical coherence tomography (OCT) revealed a reversed lenticule. The patient underwent a repeat DSAEK and had an uneventful postoperative course. The visual acuity was 20/40 after 7 months with a clear cornea and a well attached graft.

**Discussion**: PGF is a rare complication following DSAEK which occurs due to poor endothelial function of the donor graft. Insertion of a reversed lenticule may get overlooked as a cause of PGF unless the graft edge profile is examined on an OCT scan. The graft in the current case was well attached despite its inverted position suggesting that graft adherence is perhaps not a function of the corneal endothelial pumps in isolation and may be driven by factors such as the intraocular pressure.

**Conclusion**: A reversed DSAEK lenticule may have normal adherence to the host stroma and must be considered in cases with PGF. OCT of the graft edge is required for diagnosis before performing a repeat keratoplasty.

## Introduction

The advent of endothelial keratoplasties has revolutionised the treatment of corneal endothelial disorders and has enabled the restoration of a near normal corneal anatomy and physiology. ^1^^,^^2^ Also, the complications associated with a conventional penetrating keratoplasty (PK) such as suture related infections, vascularization and risks of an open sky procedure are drastically decreased with these lamellar surgeries. ^1^^,^^2^ However, these procedures are not completely risk free and suboptimal results can ensue from graft detachment, rejection, chronic endothelial attrition, etc. ^1^^,^^3^ Primary graft failure is one such complication that can occur following Descemet stripping automated endothelial keratoplasty (DSAEK) and is defined as corneal stromal edema which does not clear despite the presence of a well apposed graft. ^1^^,^^3^ A donor graft with decreased endothelial count or loss of endothelial cells due to traumatic surgical manoeuvring are the most common causes of primary graft failure. This intraoperative manipulation of the graft is done primarily to ensure the correct orientation of the lenticule. Although several approaches exist to facilitate the same, it may still get attached in an inverted fashion, especially with very thin lenticules or in eyes with significant corneal scarring. ^4^^,^^5^ Insertion of an inverted graft has not been reported as a cause of primary failure and so this report intends to describe the clinical features and management of primary graft failure in an eye with an attached yet reversed DSAEK lenticule.

## Case presentation

**Figure 1.**
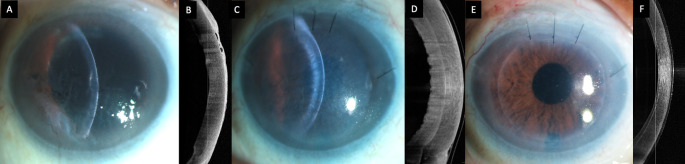
(A, B) Slit lamp image of the right eye at presentation depicting corneal edema with Descemet membrane folds and peripheral bullae which are also visible on the optical coherence tomography scan (OCT). ( **C, D) Image captured one month after the Descemet stripping automated endothelial keratoplasty (DSAEK) showing primary graft failure with a well attached graft. (E, F) Clear cornea with a attached DSAEK lenticule 2 weeks after the repeat endothelial keratoplasty.**

A 66-year-old gentleman had complaints of intermittent pain and watering in the right eye after having undergone a small incision cataract surgery with a posterior chamber intraocular lens twelve years before presenting to us. This complaint had exaggerated in the three months prior to presentation and was now associated with a decrease in vision. There was no past history of any prior interventions, and the patient did not give any relevant family history. At presentation, his visual acuity in the right eye was counting fingers, while that in the left eye was 20/25. Slit lamp examination revealed an edematous cornea with epithelial bullae in the periphery ( Figure 1). The anterior chamber was hazily seen and appeared normal with a stable intraocular lens. The posterior segment was hazily visible and revealed no abnormalities. The left eye had a visual acuity of 20/20 with a clear cornea and no guttate changes. A cataractous lens was present. Rest of the anterior and posterior segment examination was within normal limits. Specular microscopic examination revealed a healthy endothelial morphology and count in the left eye. A diagnosis of pseudophakic bullous keratopathy was made and the patient was planned for a DSAEK.

**Figure 2.**
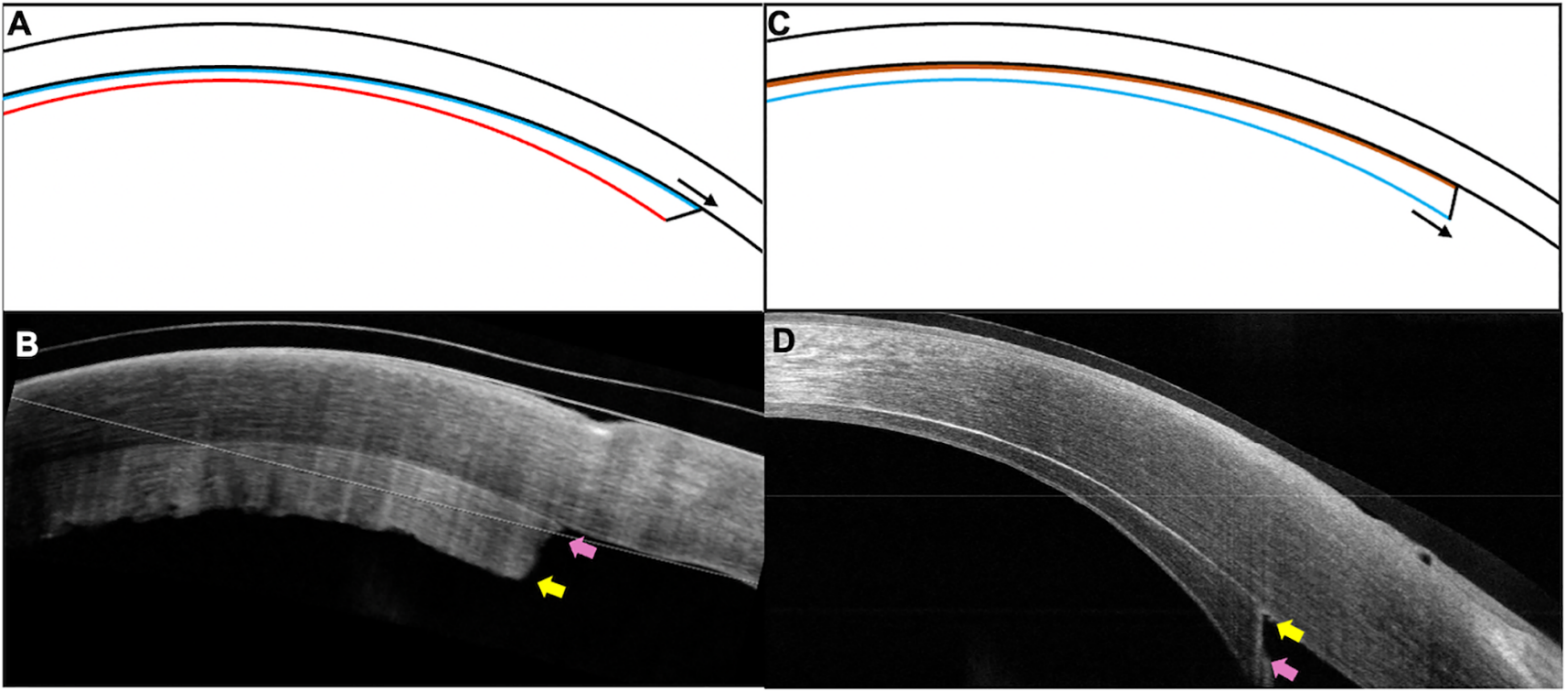
(A, B) Schematic image and the corresponding clinical picture of the inverted lenticule showing the longer endothelial side (blue line, pink arrow) towards the host stroma which has the irregular donor stromal surface (red line, yellow arrow) facing the anterior chamber (C, D) Graft attached in its correct orientation with the endothelial side facing the anterior chamber with its edge extending beyond the edge of the stromal surface (black and pink arrows).

The surgery was carried out under local anesthesia and followed a standard technique. ^6^^–^^8^ Briefly, the central 9 mm of the host Descemet’s membrane was stripped. An 8mm trephination was carried out on a pre-cut donor tissue with an endothelial count of 2508 cells/mm ^2^. The graft was inserted with a Sheets glide and apposed to the host stroma with a full chamber air tamponade of ten minutes. Postoperatively the visual acuity was counting fingers and the graft was edematous but well attached on the optical coherence tomography (OCT) scan ( Figure 1). The patient was started on topical corticosteroids (prednisolone acetate 1%, six times/day) and antibiotics (moxifloxacin 0.5%, 4 times/day). The patient was reviewed again after three weeks and no improvement in either the visual acuity or the corneal clarity was noted. The OCT was repeated and the graft edge was included in the scan which showed a reversed orientation of the graft with the longer endothelial side adherent to the stroma ( Figure 2). Furthermore, the irregular donor stromal surface facing the anterior chamber was also observed ( Figure 2).

**Figure 3.**
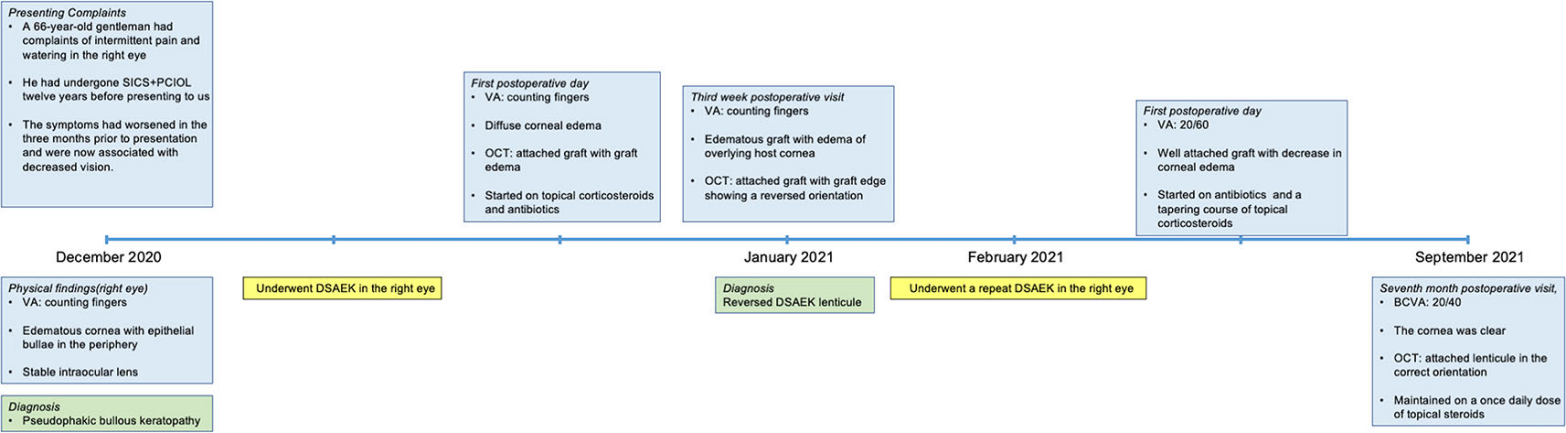
Timeline of the patient from presentation to the last follow up with details of the clinical features, interventions and outcomes. SICS: small incision cataract surgery, PCIOL: posterior chamber interocular lens, DSAEK: Descemet stripping automated endothelial keratoplasty, OCT: optical coherence tomography, VA: visual acuity.

The patient underwent a repeat DSAEK, 6 weeks after the primary surgery. After the previous donor tissue was detached and removed, a new DSAEK lenticule, 7.5 mm in diameter, with an endothelial count of 2457 cells/mm ^2^ was inserted. The rest of the surgical technique was similar to that of the first procedure. On the first postoperative day, the patient had a visual acuity of 20/60 with a well attached graft and a significant decrease in the corneal edema. The correct apposition and orientation of the graft was confirmed on OCT ( Figure 2). The patient was continued on topical antibiotics and a tapering dose of topical corticosteroids. At the 7 ^th^ month postoperative visit, the corrected visual acuity was 20/40 in the right eye. The cornea was clear with an attached lenticule, and the patient was maintained on a once daily dose of topical steroids ( Figure 1). Figure 3 details the timeline of the patient from presentation to the last follow up.

## Discussion

Several studies have investigated the factors that affect graft attachment following a DSAEK. ^5^^,^^9^^,^^10^ One of the key elements that facilitates good adherence of the lenticule is the endothelial pump action. ^9^ A well-functioning endothelium also helps to clear the corneal edema. Hence primary graft failure and graft detachment is observed in eyes with a poor quality donor tissue or excessive intraoperative manipulation. ^1^^,^^3^ Dislocation of the graft in such cases is probably mediated by the presence of fluid in the interface, which the dysfunctional endothelium is unable to overcome. And so, various modifications of the DSAEK technique have been employed which provide an additional means for graft apposition until the functional pumps can take over. These include surface massage to displace the interface fluid, use of venting incisions and roughening of peripheral stromal fibers to create stronger adhesions. ^5^^,^^9^^,^^10^

The use of air tamponade and increase in intraocular pressure (IOP) has also been described for the same purpose. Vaddavalli et al and Bhogal et al independently studied the role of IOP in graft adherence. ^5^^,^^10^ Both studies did not find a significant correlation between the two although Vaddavalli et al found an increasing rate of graft attachment with higher IOP. However, the experimental set up was devised to test the attachment under normal and high pressures and the role of hypotony was not assessed. Thus, IOP probably significantly contributes to good attachment of the donor lenticule as seen in the current case where a well attached graft was seen despite its inverted position. This also highlights the fact that there may be several other factors besides the endothelial pump action which bring about the binding of the two stromal surfaces. This needs to be taken into consideration especially in eyes which have a higher rate of graft dislocation such as aphakic eyes, vitrectomised eyes or eyes with glaucoma filtering surgery. ^1^^,^^3^ Ensuring a watertight globe at the end of the surgical procedure will help prevent postoperative hypotony and decrease the risk of graft detachment.

Primary graft failure is a rare complication following DSAEK, with a reported rate of around 1%. ^3^ The diagnosis of this entity is made in the presence of persistent corneal edema following a DSAEK. The normal time taken for the recovery of graft clarity following DSAEK ranges from three weeks to three months which can cause a delay in establishing the diagnosis of primary failure. ^3^ As a result, the subsequent interventions required to visually rehabilitate the patient will also get stalled. However, a reversed lenticule is an exception to this as it can be detected in the early postoperative period and promptly managed. Several measures have been advocated to help ensure the insertion and attachment of the graft in its correct position. These include marking of the stromal surface, use of the double ring sign, intraoperative OCT, etc. ^4^^,^^11^ However, the lenticule may get inverted despite these safeguards especially in cases with very thin grafts or in eyes with suboptimal clarity of the anterior chamber structures due to corneal scarring.

As highlighted by the current case, judicious use of an OCT in the weeks following the surgery can help identify the improper orientation of the lenticule in eyes with slow recession of corneal edema. Although documentation of an attached lenticule with an AS-OCT scan is a routine practice following endothelial keratoplasty, it is essential to include the peripheral graft area when capturing such images. An acute angle configuration of the graft edge has been described which indicates a stroma-to-stroma apposition with correct positioning of the endothelial layer. ^4^ This additional information is vital as the mere presence of an attached lenticule does not signify its correct orientation.

## Conclusion

The current report highlights a reversed DSAEK lenticule as a cause of primary graft failure. This clinical entity can be diagnosed early with the help of an AS-OCT in eyes with slow resolution of the corneal edema and a repeat intervention can be planned subsequently. Adhesion of the graft in DSAEK may depend on factors beyond functional endothelial pumps and an improved understanding of the same will help improve outcomes in eyes which are at a greater risk of having graft detachments.

## Data availability

All data underlying the results are available as part of the article and no additional source data is required

## Reporting guidelines

Figshare. CARE guidelines. DOI: https://doi.org/10.6084/m9.figshare.20501754. ^12^

## Consent

Written informed consent was obtained from the patient for publication of this case report and accompanying images.

## Author contributions

SYB contributed to the study conceptualization, study investigation, methodology, manuscript editing and revision. AK contributed to the original draft preparation, study methodology and revisions.
