# Supplementary material for: Case Report: Primary graft failure due to a reversed lenticule in Descemet Stripping Automated Endothelial Keratoplasty
Source: F1000Res. 2023 Jan 6;11:1105. Originally published 2022 Sep 27. [Version 2] doi: 10.12688/f1000research.123313.2 (PMC9627103; doi:10.12688/f1000research.123313.2)
Supplement: Peer review history

# Peer review: Reviewer response for version 1

**DOI:** 10.5256/f1000research.135408.r152987

**Published:** 2022-11-01

**Authors:** Denise Loya Garcia

**Affiliations:** 1 Institute of Ophthalmology and Visual Sciences, School of Medicine and Health Sciences, Tecnologico de Monterrey, Monterrey, Mexico; 2 Instituto de Oftalmología Fundación Conde de Valenciana I.A.P, Mexico City, Mexico

The authors describe an interesting case of Primary graft failure due to reversed lenticule in Descemet Stripping Automated Endothelial Keratoplasty and a very valuable use of the anterior segment OCT.

 Slit-lamp biomicroscopy may not be as precise in edematous corneas as details may be easily missed, even by expert clinicians. Anterior Segment Tomography is a very useful tool in searching for clinical cues that may guide the clinician in the diagnosis. As clearly demonstrated by the authors in this case report, OCT of the graft edge may demonstrate the reversed position of the lenticule a detail that would be overlooked by biomicroscopy.

Are enough details provided of any physical examination and diagnostic tests, treatment given and outcomes?

Yes

Is the case presented with sufficient detail to be useful for other practitioners?

Yes

Is sufficient discussion included of the importance of the findings and their relevance to future understanding of disease processes, diagnosis or treatment?

Yes

Is the background of the case’s history and progression described in sufficient detail?

Yes

Reviewer Expertise:

Cornea, Corneal Transplant.

I confirm that I have read this submission and believe that I have an appropriate level of expertise to confirm that it is of an acceptable scientific standard.

# Response

**Published:** 2023-01-04

**Authors:** Sayan Basu

**Affiliations:** L V Prasad Eye Institute, Hyderabad, India

We thank the reviewer for their comments

# Peer review: Reviewer response for version 1

**DOI:** 10.5256/f1000research.135408.r151990

**Published:** 2022-11-01

**Authors:** Jaime D. Martinez

**Affiliations:** 1 Anne Bates Leach Eye Center, Bascom Palmer Eye Institute, Department of Ophthalmology, University of Miami Miller School of Medicine, Miami, FL, USA

**Summary**

 Interesting paper. This case presents a patient who had primary graft failure after a DSAEK graft. Graft failure was secondary to reverse graft tissue. The patient had a repeat DSAEK graft after surgery with the correct corneal graft position with a successful outcome. I like how they describe what to look for in these specific situations on OCT images to confirm the diagnosis. I think this is important as many cases are difficult to distinguish if the graft is in the correct position even with the reference maker.

 It will be helpful for the surgeon to picture the different findings on OCT by doing the following:

- Add in the Figure 2 legend the different types of angle configurations between cases. Maybe consider adding a geometrical line on the edges with acute and obtuse angles on OCT images.

Are enough details provided of any physical examination and diagnostic tests, treatment given and outcomes?

Yes

Is the case presented with sufficient detail to be useful for other practitioners?

Yes

Is sufficient discussion included of the importance of the findings and their relevance to future understanding of disease processes, diagnosis or treatment?

Yes

Is the background of the case’s history and progression described in sufficient detail?

Yes

Reviewer Expertise:

Ophthalmology

I confirm that I have read this submission and believe that I have an appropriate level of expertise to confirm that it is of an acceptable scientific standard.

# Response

**Published:** 2023-01-04

**Authors:** Sayan Basu

**Affiliations:** L V Prasad Eye Institute, Hyderabad, India

We would like to thank the reviewer for reading and commenting on our submission.

 The required change has been made to the figure 2

# Peer review: Reviewer response for version 1

**DOI:** 10.5256/f1000research.135408.r151994

**Published:** 2022-10-25

**Authors:** Ka Wai Kam

**Affiliations:** 1 Department of Ophthalmology & Visual Sciences, The Chinese University of Hong Kong, Hong Kong, China

The authors reported a patient who underwent DSAEK and developed persistent corneal oedema after transplantation despite graft adherence. 

 In addition to the use of ASOCT, an attentive slit lamp examination with a narrow light beam and focusing on the graft edge may alert the clinician about a potential graft reversal. This may be highlighted as ASOCT may not be readily available in every eye centre. 

 In the report, the authors mentioned the use of Sheets glide in implanting the lenticule. Unlike in DMEK where some surgeons would use an S-stamp or an asymmetrical triangle mark at the graft periphery, graft orientation in DSAEK relies on a careful preparation and insertion process. It would be interesting to compare the different insertion techniques (Sheets glide, Busin glide, Endoglide) and look at the rate/risk of graft lenticule reversal - but in reality, there are multiple factors that could interfere with the final outcome.

 Similarly, the authors may wish to elaborate on what in their opinion contributed to the graft reversal during the first transplantation, and what special precaution was taken during the second transplantation in order to avoid the same complication. This would be beneficial to novice EK surgeons in ensuring a correct graft insertion. 

 In places where grafts are scarce, reversal of a flipped endothelial keratoplasty may be attempted if the reversal had been noted earlier. In our experience, we had treated two flipped DSAEK buttons on postoperative day 1 by reversing the graft in the operating theatre. One of the two grafts cleared up after the reversal. This option may be considered especially in areas where corneal grafts are not as abundant.

Are enough details provided of any physical examination and diagnostic tests, treatment given and outcomes?

Yes

Is the case presented with sufficient detail to be useful for other practitioners?

Yes

Is sufficient discussion included of the importance of the findings and their relevance to future understanding of disease processes, diagnosis or treatment?

Yes

Is the background of the case’s history and progression described in sufficient detail?

Yes

Reviewer Expertise:

Cornea

I confirm that I have read this submission and believe that I have an appropriate level of expertise to confirm that it is of an acceptable scientific standard.

# Response

**Published:** 2023-01-04

**Authors:** Sayan Basu

**Affiliations:** L V Prasad Eye Institute, Hyderabad, India

1. In addition to the use of ASOCT, an attentive slit lamp examination with a narrow light beam and focusing on the graft edge may alert the clinician about a potential graft reversal. This may be highlighted as ASOCT may not be readily available in every eye centre. 

*We thank the reviewer for their comment and agree that slit lamp examination can provide an understanding of the graft orientation especially in eyes with limited scarring and a good view of the anterior chamber. This point has been added to the discussion.*

 2. In the report, the authors mentioned the use of Sheets glide in implanting the lenticule. Unlike in DMEK where some surgeons would use an S-stamp or an asymmetrical triangle mark at the graft periphery, graft orientation in DSAEK relies on a careful preparation and insertion process. It would be interesting to compare the different insertion techniques (Sheets glide, Busin glide, Endoglide) and look at the rate/risk of graft lenticule reversal - but in reality, there are multiple factors that could interfere with the final outcome.

*We concur with the reviewer that understanding the rates of graft reversal in different insertion techniques will help identify the one associated with the least risk of graft reversal and this procedure can be adopted in eyes where in a higher risk of reversal is anticipated. This has been added to the discussion.*

 3. Similarly, the authors may wish to elaborate on what in their opinion contributed to the graft reversal during the first transplantation, and what special precaution was taken during the second transplantation in order to avoid the same complication. This would be beneficial to novice EK surgeons in ensuring a correct graft insertion *.*

*The first procedure was performed by a surgeon with relative inexperience and marking of the graft was not carried out. This could have resulted in the inversion of the graft during its insertion. This point along with measures to prevent the same has been added to the discussion to help surgeons early in the course of their training.*

 4. In places where grafts are scarce, reversal of a flipped endothelial keratoplasty may be attempted if the reversal had been noted earlier. In our experience, we had treated two flipped DSAEK buttons on postoperative day 1 by reversing the graft in the operating theatre. One of the two grafts cleared up after the reversal. This option may be considered especially in areas where corneal grafts are not as abundant.

*We agree that attempting a graft reversal is a viable option in low resource settings provided a reasonable time interval exists between the primary EK and the secondary procedure. This point has been added to the discussion.*
